# Observations on Rabies Contagiosa

**Published:** 1819-04-01

**Authors:** Daniel Johnson

**Affiliations:** formerly a Medical Officer in the Honourable East India Company's Service


*Observations on Rabies Contagiosa.* By DANIEL JOHNSON, Esq. formerly a Medical Officer in the Honourable East India Company's Service.

HAVING passed all the middle part of life in India, a country by no means friendly to European constitutions, particularly to those, who, like myself, were impatient of confinement, and regardless of the climate, I am now suffering from its baleful influence, with little expectation of relief. To amuse the tedious hours of a sick room then, I shall take up my pen, occasionally, to communicate to the author of the "Influence of Tropical Climates on European Constitutions," (a work which needs no encomiums from me) such observations as my long residence in India has enabled me to make; with the hope that they may prove useful to my countrymen in that distant quarter of the globe, especially when promulgated through the medium of Dr. Johnson's writings, which must have an extensive circulation between the tropics

The subject of this my first paper is Hydrophobia, a dreadful, and, I believe, hitherto an incurable disease. The number of persons bitten by mad dogs and mad jackals, that came under my care, while surgeon at Chittrah, (Rhumghur) would appear almost incredible, were they to be stated here.

In *every instance* where I had time or permission to impregnate the system with mercury after the infliction of the bite, and before the symptoms of hydrophobia shewed themselves, the latter were entirely prevented. If it be feared that I may have been deceived on this point, I hope to dissipate such fears by stating that not a year passed at the station abovementioned, in which I had not numbers to attend to, *bitten by the same animal.* Of these there were some who, from religious prejudices, would not submit to the course of medicine I prescribed, preferring the prayers of the Brahmin priest. *These* regularly perished by the disease, while the others, bitten by the same animal, and at the same period of time, were invariably preserved from hydrophobia, where salivation was induced. This, which I think may be fairly called the *experimentum crucis,* I have put to the test so often, with the same identical result, that not a shadow of doubt remains on my mind relative to the entire efficacy of the prophylactic. The proofs, indeed, are positive, negative, and comparative; and I leave it to the consideration of the profession at large, and especially of those employed in our Indian territories, where the occurrence of hydrophobia is so frequent, whether or not to adopt a preventive measure which offers so certain a check to this most dreadful of all diseases. Neither salivation nor any other remedy has ever, in my experience, arrested the disease, when once it had commenced its destructive career.

It often happens that mad dogs or jackals get into the kennels or dog houses in India, and sometimes even mingle with the dogs in the field while sporting. This is when they are in the first stage of madness, and they will then go considerably out of their way to attack and bite all that come in their sight. In such cases a general examination must be made, and every dog that bears the least mark of scratch or bite must be put to death. Even this precaution does not always ensure perfect safety, as the following, selected from several other facts, will tend to show. While I

was coursing one day with a leash of greyhounds and four or five terriers, a jackal appeared at a considerable distance on a plain. The greyhounds were slipped; the dogs saw the animal, and immediately made directly for him. To my great surprise, the jackal, instead of making off, ran straight towards the dogs, and I soon discovered that he was raging mad. It was impossible to separate them till they had killed him. I went immediately home, had all the dogs washed, and examined them myself in the most minute manner. I found four favourite dogs bitten, and these were instantly hanged. The others, having no marks of the least scratch, I considered as safe. About three weeks afterwards, however, on my march to Calcutta, my dog-keeper came running up to my tent, one day, crying aloud, and at the same time keeping three terriers, as well as he could, at arm's length, they making all possible efforts to bite him. As soon as he approached, I saw, by their erect hair, like bristles, inflamed eyes, and foaming mouths, that they were mad; I therefore directed the poor fellow to twist their cords round a tree, which he dexterously effected, and then I caused them to be dispatched with a wooden mallet, used for driving the tent pins. The dog-keeper was bitten in at least twenty places; some of them trifling, others large bites. To the whole of these I applied lunar caustic, and put him into a salivation as quickly as I could. The ptyalism was kept up for fourteen or fifteen days. He lived with me several years afterwards, and remained in perfect health.

On another occasion I had a small pet spaniel puppy, about six months old, tied up in the verandah, which cried out violently, one day, as if something was killing it. On the servant's running to see the cause, a hyena threw it out of his mouth, and very reluctantly went off. The puppy was washed and minutely examined, but no injury could be discovered. The puppy was, however, smeared over with slime, which must have been the saliva of the hyena. No idea was entertained at the time that the hyena was mad, though he certainly quitted the premises with more reluctance than is commonly observed. About three weeks after this, the puppy came running into a room where nearly fifty people were assembled at a notch, or Hindostannee dance, raging mad. The little creature instantly attacked every thing that came in his way, and the whole notch was instantly dispersed in all directions. Several chairs were broken before the rabid animal could be killed.

Whether, in these instances, the dogs received the poison by some of the saliva of the mad animals passing into their mouths, or by respiring the effluvia arising from them, I cannot take upon me to say; but I can confidently assert, that they had no wounds. The above will satisfy gentlemen that, after a dog has been worried, or come in contact with another that is mad, he should be kept tied up for a month, to see the event. I may here state an important fact which I had ample means of unequivocally ascertaining; namely, that in no one instance, did a dog become mad, after remaining well after the bite a month. The usual period, in India at least, and as far as came under my own observation, was from fourteen to twenty-five days after the reception of the poison.

There is a generally received opinion in India, that dogs and jackals become more frequently mad there, in consequence of the number of putrid human carcases which they have to feed on. But this idea is, I think, erroneous; because, at Chittrah, rabid animals are as common as in any part of India, or perhaps more so; yet, in that place, no human carcase is to be seen, in consequence of the abundance of fuel to be procured for nothing, which enables the inhabitants to burn their dead—a ceremony from which the Hindoos are only prevented in any place by scarcity of fuel. I may remark another curious circumstance which I have repeatedly and invariably observed, namely, that the animals abovementioned, are most frequently mad at the time the jungle fever is most prevalent, and *vice versa*.

Finally, may I be permitted to object to the term HYDROPHOBIA; since I have very generally observed that, in the incipient stage of the disease, instead of *dread*, there is the most intense thirst and craving *desire for water*. The power of swallowing the fluid, however, is taken away; and every attempt brings on a violent and painful spasm of the diaphragm and muscles of deglutition and respiration. It is, I am convinced, the dread of such painful recurrences that produces, by association of ideas and feelings, the dread and horror of water, or even the sight or name of that liquid.

D. JOHNSON.

Torrington, Devon, Dec. 1818.

*To Dr. James Johnson.*

---

☞ The Editor has omitted the very well drawn up *Cases* of Rabies Contagiosa, accompanying the above valuable paper, as they only depict the usual features of that terrible disease. The paper itself will be read with no common interest; and the prophylactic measure, which rests on such a firm basis of experience—an experience which can fall to the lot of few individuals, must be duly appreciated by an enlightened profession.

Editor.

